# Latest Advances in the Use of Therapeutic Focused Ultrasound in the Treatment of Pancreatic Cancer

**DOI:** 10.3390/cancers14030638

**Published:** 2022-01-27

**Authors:** Petros X. E. Mouratidis, Gail ter Haar

**Affiliations:** Department of Physics, Division of Radiotherapy and Imaging, The Institute of Cancer Research: Royal Marsden Hospital, Sutton, London SM25NG, UK; gail.terhaar@icr.ac.uk

**Keywords:** HIFU, histotripsy, therapeutic focused ultrasound, pancreatic cancer, immunotherapy

## Abstract

**Simple Summary:**

Pancreatic cancer is a devastating disease with the worst survival rates of all cancers. Advances in immunotherapy have revolutionized the treatment of cancer, providing cures to patients with a previously lethal disease. Sadly, clinical trials of currently established immunotherapies have not provided significant therapeutic benefits to pancreatic cancer patients. For this reason, alternative treatment approaches that could sensitise the pancreatic tumours to immunotherapy are needed. Therapeutic focused ultrasound is a non-invasive physical modality that can kill cells by raising the temperature, and by disrupting and destroying tissue mechanically. It can also sensitise tumours to the effects of radiotherapy. This review article describes the use of therapeutic focused ultrasound in the treatment of pancreatic cancer. Attention is given to the immunological effects induced by therapeutic focused ultrasound, and how these can be used to sensitise the pancreatic tumours to immunotherapy.

**Abstract:**

Traditional oncological interventions have failed to improve survival for pancreatic cancer patients significantly. Novel treatment modalities able to release cancer-specific antigens, render immunologically “cold” pancreatic tumours “hot” and disrupt or reprogram the pancreatic tumour microenvironment are thus urgently needed. Therapeutic focused ultrasound exerts thermal and mechanical effects on tissue, killing cancer cells and inducing an anti-cancer immune response. The most important advances in therapeutic focused ultrasound use for initiation and augmentation of the cancer immunity cycle against pancreatic cancer are described. We provide a comprehensive review of the use of therapeutic focused ultrasound for the treatment of pancreatic cancer patients and describe recent studies that have shown an ultrasound-induced anti-cancer immune response in several tumour models. Published studies that have investigated the immunological effects of therapeutic focused ultrasound in pancreatic cancer are described. This article shows that therapeutic focused ultrasound has been deemed to be a safe technique for treating pancreatic cancer patients, providing pain relief and improving survival rates in pancreatic cancer patients. Promotion of an immune response in the clinic and sensitisation of tumours to the effects of immunotherapy in preclinical models of pancreatic cancer is shown, making it a promising candidate for use in the clinic.

## 1. Introduction

### 1.1. Pancreatic Cancer

Despite advances in molecular biology and immunotherapy, no significant improvement in prognosis for pancreatic cancer patients has been seen over the past 30 years. Tumours in the pancreas begin as pancreatic intraepithelial neoplasias (PanIN), and subsequent mutations cause them to progress to tumours [1,2,3]. The most frequent mutations include the oncogenic Kirsten Rat Sarcoma Viral Oncogene Homolog (KRAS) mutations [1], tumour suppressor Tumour Protein 53 (TP53) inactivating mutations [4], Cyclin-dependent Kinase Inhibitor 2A mutations which lead to dysregulation of the cell cycle [5], and inactivating mutations of the tumour suppressor Deleted in Pancreatic Cancer 4, which occur in approximately 90, 70, 50 and 40% of pancreatic tumours, respectively [6]. Less frequent mutations can be seen in epigenetic or other regulatory genes [7,8,9,10]. The majority of the resulting primary pancreatic tumours are pancreatic ductal adenocarcinomas located in the head of the pancreas, and metastasis can occur before their enlargement and detection [11,12]. Thus, patients present late for diagnosis with the disease, at an advanced stage. This results in only ~20% of patients being eligible for surgery. Surgery of the primary tumour is the only curative option, but even in these cases, patients may often relapse.

Radiotherapy, chemotherapy and immunotherapies have all failed to improve survival, resulting in the worst 5-year prognosis of all common malignancies [13]. 5-fluorouracil (5-FU) was the standard chemotherapeutic drug for treating pancreatic cancer until 1997. Gemcitabine then became the 1st-line treatment for patients with locally advanced, metastatic disease as it was shown to offer better survival than 5-FU (1-year survival of 18% versus 2%), and fewer side effects [14]. Gemcitabine monotherapy remained the preferred treatment option until 2011 when PDAC patients receiving FOLFIRINOX, a combination of oxaliplatin, irinotecan and 5-FU, achieved a median overall survival of 11.3 months vs. 6.8 months when compared to the gemcitabine-only group [15]. For older patients, or those with high disease burden and borderline performance status the combination of gemcitabine with nab-paclitaxel can also be used because it offers fewer side effects than FOLFIRINOX [16,17].

One reason for the failure of treatments to provide therapeutic benefit in these patients has been postulated to be the low immunogenicity of pancreatic tumours due to their lack of neoantigens. This is because pancreatic carcinogenesis is driven by a low number of mutations typically ~3 mutations/Megabase [6,18]. Another reason is their dense fibrotic stroma which makes up to 90% of the tumour mass [19], increases the tumour’s interstitial pressure [20], collapses the vasculature [21,22] and decreases the penetration of drugs and effector immune cells [23,24]. In addition to the collagen and hyaluronan-rich matrix, pancreatic stroma consists of several cell subtypes, and crosstalk between them and the tumour cells regulates tumour growth and resistance to treatments [25,26,27]. The stroma contains pancreatic stellate cells (PSC) which regulate the tumoural microenvironment [28,29], cytokine and chemokine-responsive tumour associated macrophages (TAM) which contribute to desmoplasia and immunosuppression [30,31], T_regulatory_ cells, myeloid-derived suppressor cells (MDSC) which build crosstalk with TAM and suppress the effector T cells [32,33] and cancer-associated fibroblasts (CAF) [34]. The latter are differentiated from resident fibroblasts, activated PSCs, adipocytes and tumour-infiltrating mesenchymal stem cells [35], and can create an immunosuppressive microenvironment [36,37,38,39,40,41,42,43].

Clinical efforts to tackle pancreatic cancer by depleting the pancreatic tumour microenvironment have failed so far [44,45,46], leading to the suggestion that the tumour microenvironment may have an anti-tumour role to play in at least some stages of disease progression [25,26]. The need for a more targeted approach against the pancreatic tumour microenvironment has therefore become clear and recently, improved response rates in patients with high levels of hyaluronic acid in their tumours, have been obtained from a phase I/II study (NCT01839487) which combined a high molecular weight hyaluronan inhibitor PEGPH20 with gemcitabine [47].

### 1.2. Immunotherapy

**Figure 1:**
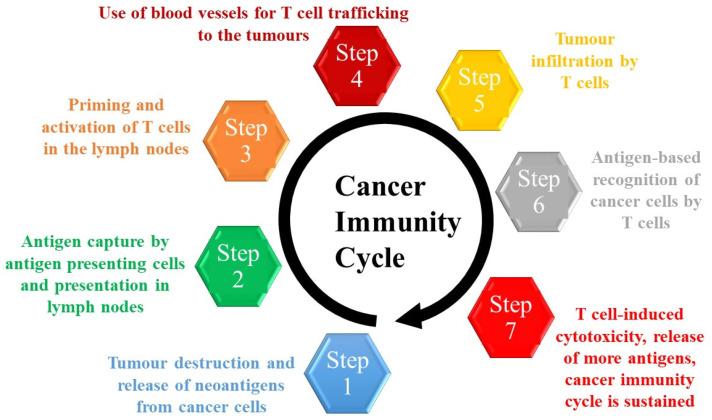
The Cancer Immunity Cycle.

The cancer immunotherapy revolution has led to treatments that can provide clinical benefits in previously untreatable diseases. Several immunotherapy approaches, including immune checkpoint inhibitors (ICIs) [48,49,50,51,52], have been introduced into the clinic with the aim of augmenting a self-sustaining cycle of cancer immunity known as “the cancer immunity cycle” [53] (Figure 1). ICIs have shown efficacy in immunologically “hot” tumours. In a phase III trial, 20% of melanoma patients who received Ipilimumab, a CTLA-4 inhibitor, had a better outcome than those who received control arm chemotherapy, and survived longer than 3 years, with a survival of 10 years noted for some patients [54,55]. In 2011 this inhibitor received FDA approval for the treatment of metastatic melanoma and in 2014, approval of PD-1 ICI followed [56,57,58]. The appeal of ICIs is that these successes have been reproduced in other tumour types, and so far, these antibodies have been FDA-approved for the treatment of more than 50 indications, including melanoma, lung, genitourinary, head and neck, breast, lymphoma, gynaecological and gastrointestinal cancers. They are also approved for patients with microsatellite instability-high and/or mismatch-repair deficiency tumours [59,60], and all high mutational burden tumours, including pancreatic tumours [61]. The combined blockade of CTLA4 and PD-1/PD-L1 has also resulted in higher response rates than when either are used as a monotherapy in several cancer types [62,63,64,65,66,67,68,69,70,71]. The efficacy of immunotherapies is limited by their toxicity and by intrinsic (immune recognition, cell signalling, gene expression, and DNA damage response) and extrinsic (impaired T cell activation) cell resistance mechanisms. For these reasons response rates to ICIs have been limited and some patients who initially respond will ultimately have disease progression [72]. For this reason, efforts are underway to discover new treatment modalities that could render immunologically “cold” tumours “hot”, prime the immune system and the cancer immunity cycle, and improve the penetration of immune therapeutics into tumours.

### 1.3. Therapeutic Focused Ultrasound

**Figure 2:**
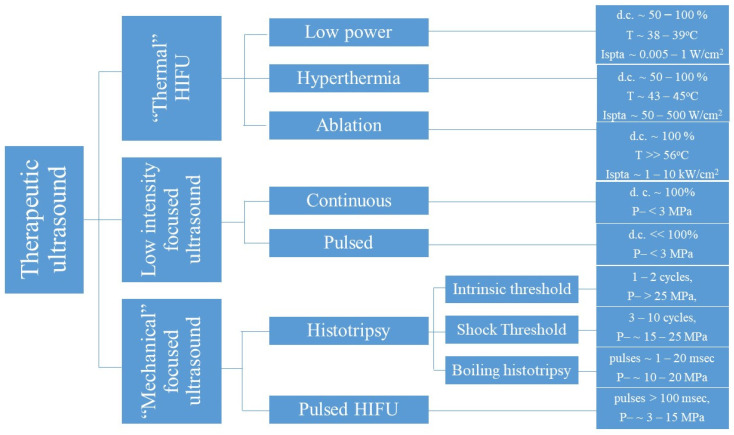
Therapeutic focused ultrasound can induce both “thermal” and “mechanical” effects on tissues.

Therapeutic focused ultrasound is a physical modality that can induce localised thermal and mechanical effects non-invasively due to the compressions/decompressions induced when tissue is subjected to acoustic pressure waves [73]. The important parameters for describing ultrasound exposures include the frequency of the ultrasound wave (f), the pulse repetition frequency (prf), duty cycle (d.c.), duration, power, pressure and intensity (spatial average temporal average (I_sata_) or spatial peak temporal average (I_spta_). There is an interplay between these parameters that determines whether thermal or mechanical effects predominate. At low frequencies and high-pressure amplitudes, mechanical effects (specifically acoustic cavitation) can be readily induced, whereas for higher frequencies and longer pulses thermal effects predominate. When focused ultrasound is applied in its continuous “thermal” mode (exposures lasting seconds), the tissue is subjected to frictional forces which are converted to heat [74]. Hyperthermia treatments (characterised by temperatures of 43–45 °C maintained for up to an hour) have been used to sensitise tumours to the effects of radiotherapy and chemotherapy [75,76,77,78,79,80,81,82,83,84,85,86,87]. At higher power, high-intensity focused ultrasound (HIFU) heats cells rapidly, to temperatures over 56 °C for a few seconds, resulting in thermal necrosis and instantaneous cell death. The resulting “lesion” is ellipsoidal, with a sharp boundary between the treated and non-treated regions of the tissue [78,79,88,89] (Figure 2).

Histotripsy is the term used to describe focused ultrasound exposures that consist of high amplitude peak negative pressures (P–) (>15 MPa) applied to the tissue for milliseconds or less with the intention of inducing mechanical damage without significant temperature rises [90,91,92,93]. Three forms of histotripsy have been identified, depending on the number of acoustic cycles used and the P– amplitudes required: intrinsic threshold histotripsy (1–2 cycles, P– > 26 MPa), shock scattering histotripsy (3–10 cycles, P– = 15–25 MPa) and boiling histotripsy (longer 1–20 ms pulses, P– = 10–20 MPa) [94]. In addition to histotripsy, pulsed HIFU (pHIFU) exposures can also be used to permeabilize and/or destroy tissue using lower P– (<10 MPa) and longer exposure times (>> milliseconds) than histotripsy. The effects of pHIFU are predominantly mechanical with duty cycles designed so that the “Off time” (typically between 1–20%) is long enough to minimise heat accumulation. The effects of “mechanical” focused ultrasound result from the creation of cavitation, and the application of radiation forces, “streaming” and “microstreaming” in the target tissue. Cavitation refers to the ultrasound-induced formation of gas-containing bubbles in the tissue [95,96]. These bubbles either oscillate stably or collapse violently as a result of ultrasound exposure. Their movement and/or collapse results in the permeabilisation and/or destruction of tissue. Radiation forces are those exerted on tissue by an ultrasound wave, and “streaming” and “microstreaming” refer to the creation of small-scale fluid motion near cells as a result of radiation forces or around stably oscillating cavitation bubbles, respectively. Finally, low intensity focused ultrasound (LOFU) exposures either in continuous or pulsed mode in the presence or absence of microbubble contrast agents use low-intensity exposures which promote wound healing, muscle regeneration and tissue permeabilization with therapeutic intent. LOFU exposures typically use frequencies of 1.5 MHz, spatial average temporal average intensities of 30 mW/cm^2^ and, in the case of pulsing regimes, are in the order of 200 μs/800 μs “On time/Off time” [97,98,99,100,101,102,103,104] (Table 1).

Therapeutic focused ultrasound-mediated therapy has found several applications in the clinic owing to its multiple mechanisms of action and pleiotropic downstream biological effects. So far, these treatments have received FDA approval for the treatment of essential tremor, uterine fibroids, prostate cancer, osteoid osteoma and the palliation of pain from bone metastasis. It is also being investigated for its use in more than 150 clinical indications or disorders [107]. Recently the immunological effects of therapeutic focused ultrasound have received increased attention. The purpose of this review is to detail the most important advances in ultrasound-induced immunity with a particular focus on the field of pancreatic cancer research. Both “thermal” and “mechanical” focused ultrasound can initiate immune responses in tumours by releasing tumour-associated antigens that lead to T cell-specific responses [108], increased tumour infiltrating lymphocytes (TIL) [109] and antigen-presenting cells (APC) [110] in the tumour microenvironment, activation of dendritic cells (DC) [111], change in the immune contexture of tumours [112], induction of the abscopal effect [108] and reversal of T cell anergy and tolerance [113].

## 2. Immune Effects of Therapeutic Focused Ultrasound

### 2.1. Thermal Ablation

The development of ultrasound and magnetic resonance imaging (MRI)-guided clinical systems capable of thermally ablating tissue led to the extensive investigation of the immunogenic effects of “thermal” focused ultrasound in preclinical and clinical studies (Table 2). In vitro heating of colon, lung and ovarian cells with ablative and sub-ablative thermal iso-effective doses (TID) have been shown to result in the induction of immunogenic cell death (ICD) and the release of damage-associated molecular patterns (DAMPs), including calreticulin ATP and HSP70 release, HSP70 and HSP90 exposure on the plasma membrane and CD47 downregulation (***parameters:*** *T = 45–56 °C, TID = 60 to >> 240 CEM_43_*) [80,114]. In vivo, treatment of murine hepatocellular tumours with “thermal” ablative focused ultrasound using a 2-cm-diameter transducer has doubled the cytotoxic activity of T cells, increased the secretion of IFNγ and TNFα [115] and produced a statistically significant maturation of DC and subsequent activation of T_cytotoxic_ cells against the tumours (***parameters:*** *f = 9.5 MHz, Power = 5 W, exposure time = 220 s*) [111]. In B16 melanoma tumours heating of a small region of 2–3 mm in diameter within tumours larger than 5 mm in diameter with a 16-element MR-guided HIFU annular array combined with the toll-like receptor (TLR) agonist CpG, polarised macrophages and DC towards a pro-immune phenotype, and enhanced intra-tumoral and draining lymph node antigen cross-presentation, and type I IFN release when compared to the CpG treatment (***parameters:*** *T > 60 °C, TID > 5000 CEM_43_*) [116]. These data show that “thermal” focused ultrasound creates a pro-immune tumour microenvironment by releasing DAMPS, activating T cells against the tumours and enhancing antigen presentation.

Some of the earliest studies to investigate the immunological effects of HIFU in the clinic were those of Feng Wu and colleagues, who in 2004, reported the use of the JC ultrasound-guided HIFU system (Chongqing Haifu Technology Co., Chongqing, China) to treat patients with osteosarcoma, hepatocellular carcinoma and renal cell carcinomas. One session of HIFU treatment was used to treat their primary tumours. Results showed blood increases of immune cells including T cells, T_cytotoxic_ and CD4^+^ T cells after HIFU treatment compared to before treatment (***parameters:*** *acoustic focal peak intensities = 5–20 kW/cm^2^*) [105]. Subsequent investigations using the same HIFU system in breast cancer patients showed that when HIFU was combined with surgery increased DC (~1.5 fold increase), macrophages (~2 fold increase), B cells (~2 fold increase) [110], lymphocytes (~2.5 fold increase), T_helper_ cells (~3 fold increase) and T_cytotoxic_ (~2 fold increase) [109] in the tumours compared to surgery alone. There was also an increase in the number of granzyme B and perforin positive TILs in the tumours of the HIFU and surgery group relative to the surgery only group, suggestive of their activation. These results showed that the pro-immune effects of “thermal” ablation shown preclinically can be replicated in the clinic and can help modulate the immune microenvironment of patients.

### 2.2. Pulsed Focused Ultrasound and Histotripsy

“Thermal” ablation of tissues can destroy cells and release cancer-associated antigens, but it is reasonable to hypothesize that at least some of these antigens may be heat-fixed from the extreme temperature rises (>56 °C) that are induced in tissues after HIFU treatment. This has led to suggestions that “mechanical” focused ultrasound may be better at releasing intact antigens than “thermal”. When the effects of “thermal” and “mechanical” focused ultrasound were combined with immunotherapy (CpG and anti-PD-1 antibodies), both induced innate immune responses. Here, FVB/n subjects carrying NDL tumour biopsies bilaterally were exposed to therapeutic focused ultrasound with the MR-guided Bruker BioSpec 7T small animal MR system. A 16-element 3 MHz annular array transducer (Imasonic SAS) was used for the sonications. Results showed that anti-immune MDSCs and FoxP3^+^ cells in the treated and distant tumours were increased by “thermal” parameters, whereas “mechanical” parameters caused a decrease in FoxP3^+^ cells and kept the MDSC levels unaltered in distant tumours (***“thermal” parameters:*** *P*– *= 3.1 MPa, d.c. = 100%, T > 60 °C, TID > 5000 CEM_43_, **“mechanical” parameters:** P*– *= 16.9 MPa, d.c. = 0.5%, duration = 5 s, 10 repeats*) [128]. Early studies of “mechanical” focused ultrasound in colon adenocarcinoma and prostate tumours showed that pHIFU treatments using an H-102 HIFU transducer (Sonic Concepts, Seattle, WA, USA) induced DC maturation and the accumulation of tumour-specific IFNγ-secreting cells, and increased T_cytotoxic_ responses (***parameters:*** *P*– *= 10–12.5 MPa, d.c. = 2%*) [117,118]. More recently, the temporal and tumour type dependence of the biological and immunological effects of “mechanical” focused ultrasound has been investigated in a series of studies by the J. Frank group [106,119,120,121]. Murine B16 melanoma and 4T1 breast cancer flank tumours exposed to pHIFU under ultrasound guidance using the Alpinion VIFU 2000 small animal treatment platform (Alpinion Medical Systems, Bothell, WA, USA) created an anti-tumour immune microenvironment by decreasing the expression of IL10, TGF-β, IL12p40, IL12p70 and increasing ICAM (B16 tumours) and IL17, CXCL10, ICAM and RANTES (***parameters:*** *P*– *~6 MPa, d.c. = 10%, prf = 5 Hz, pulses = 100*) [119]. In addition, MDSCs were decreased in both tumour types, and decreases in T_regulatory_ cells, M1 and M2 macrophages were seen in 4T1 breast cancer cells. These effects could be associated with the induction of DNA damage by reactive oxygen species generated from the creation of cytosolic Ca^2+^ transients by the ultrasound treatments [120]. In a follow-up study, increases in T_h1_, T_regulatory_, T_cytotoxic_, B cells, NK cells and macrophages in the TDLN of B16 and 4T1 tumours treated with the pHIFU parameters described above were seen [121]. These results showed the variability and complexity of the immune response and the difficulty in harnessing it to produce effective anti-cancer responses. Robust immune responses are necessary and for this reason, it has been proposed that histotripsy exposures able to liquefy tissue may be more appropriate for releasing intact cancer-associated antigens thus initiating the cancer immunity cycle.

To test this hypothesis, treatment of melanoma tumours engineered to express a neo-antigen-mimicking peptide GP33 with histotripsy resulted in the activation of T_cytotoxic_ in B16GP33 tumour-bearing C57BL6 animals, as indicated by increases in their intracellular IFNγ expression. The histotripsy exposure of the B16-GP33 cells was so effective at stimulating cancer-specific lymphocyte responses that their stimulation approached that of direct exposure to the GP33 peptide (***parameters:*** *f = 1 MHz, P*– *= 30 MPa, prf = 100 Hz, pulses = 50*) [108]. In addition, in vitro histotripsy treatment of the breast cancer, MDA-MB-231 cells with a 2 MHz HIFU transducer killed cancer cells, released DAMPS, including HMGB1 and HSP70, activated ICD and released pro-inflammatory cytokines, such as interferon IFNγ, members of the IL-1 family and chemokines (***parameters:*** *P*– *= 14 MPa, prf = 1 Hz, d.c. = 1%, pulse duration = 10 ms long*) [122]. In-vivo studies have shown the induction of systemic inflammation by histotripsy. In preclinical models of large immune-refractory neuroblastoma tumours, enhancement of TILs and the abscopal effect were seen after treatment of the tumours with ultrasound-guided pHIFU (***parameters:*** *f = 1.5 MHz, P*– *=14 MPa, prf = 1 Hz, pulse duration = 13.3 ms, 15 s/focus*) [123]. In renal cell carcinomas, enhanced tumoural infiltration of T_cytotoxic_ after ultrasound-guided boiling histotripsy with the VIFU-2000 platform using a 1.5 MHz single-element, spherically-focused transducer, were also seen (***parameters:*** *P*– *= 17–20 MPa, pulse duration = 10 ms, prf = 1 Hz, pulses = 10 per focus*) [124]. These results provide strong evidence for the initiation of the cancer immunity cycle by histotripsy exposures that are able to destroy tumours. The higher peak negative pressures that are required in these “mechanical” treatments compared to the “thermal” treatments led to a delay in their use in the clinic. The safety of histotripsy treatment in 25 patients with benign prostate hyperplasia has been shown in a phase I trial where treatment was performed with a transrectal investigational Vortx Rx system (HistoSonics Inc., Ann Arbor, MI, USA) that used a 700-kHz, 36-element transducer and cavitation was detected using ultrasound imaging [129].

### 2.3. Low Intensity Focused Ultrasound

Low intensity focused ultrasound (LOFU) can also produce immune effects. Their biological basis is still uncertain, and the ultrasound exposure parameters need to be fine-tuned to produce the desired effect. This is particularly important in applications in the brain where a blood–brain barrier opening in the absence of a significant immune response is required. In this context, the NF-κβ pathway-associated inflammatory response and the infiltration of macrophages in the murine brain were seen after treatment with LOFU (***parameters:*** *P*– *= 0.3 MPa, pulse duration = 10 ms, d.c. = 1%*) [126]. In a study by a different group, the magnitude of the upregulation of the proteins associated with the NF-κβ pathway was remarkably similar to that reported by Kovacs et al. [126] after exposure of the murine brain to the same focused ultrasound parameters using an MRI-guided focused ultrasound system (RK100 system, FUS Instruments Inc., Toronto, ON, Canada) [127]. Treatment of colon tumours with pulsed LOFU using an ultrasound-guided focused ultrasound exposure system (Sonic Concepts, operating frequency = 0.5 MHz, active element diameter = 64 mm, radius curvature = 55 mm) resulted in sustained tumour increases in T_cytotoxic_ and the ratio of T_cytotoxic_/T_regulatory_, 3 and 18 days after treatment when compared to controls (***parameters:*** *P*– *= 1.4 MPa, pulse duration = 100 ms, prf = 1 Hz, 20 repeats*) [125]. Furthermore, treatment of murine B16 melanoma tumours with LOFU using the Therapeutic Imaging Probe System (TIPS, Philips Research, Westchester, NY, USA) reversed T cell tolerance and anergy. These LOFU treatments overcame T cell hypo-responsiveness to tumour antigens by increasing the production of cytokines by the re-stimulated CD4^+^ T cells, and by reducing anergy-related gene expression. LOFU increased HSP70, MHCII and B7 expression, and changed the tumoural distribution of calreticulin, suggesting a pro-immunogenic effect for these treatments. Moreover, there was increased maturation/activation of DC, further supporting the hypothesis that LOFU promoted T cell activation over anergy (***parameters:*** *P*– *= 3 MPa, d.c. = 100%, I_spta_ = 550 W/cm^2^, Energy = 900 J*) [113].

Collectively, this information provides “proof of concept” that both “thermal” and “mechanical” focused ultrasound exposures can induce an immune response. The multiple physical mechanisms by which therapeutic focused ultrasound causes tissue disruption and disintegration can lead to different biological effects that may be tumour type- and tumour stage-dependent. Ultimately, our detailed understanding of these events should lead to the design of treatments aimed to create robust and durable anti-cancer immunological responses with higher anti-cancer efficiency and reduced side effects.

## 3. Treatment of Pancreatic Cancer with Therapeutic Focused Ultrasound

More than 3500 pancreatic cancer patients have been treated with palliative intent with thermally ablative HIFU in the past 20 years. Reported studies showed that not only is the treatment of pancreatic tumours with HIFU feasible and safe but also that it produces effective pain relief, and in some cases, tumour growth control, improved survival rates and favourable clinical response. In addition, the feasibility of using “mechanical” focused ultrasound and LOFU to treat pancreatic cancer is under investigation preclinically.

### 3.1. Treatment of Pancreatic Cancer Patients with “Thermal” HIFU

The earliest reports of HIFU-treated patients have come from China. In 2002 pain relief was reported in all 15 HIFU-treated patients (FEP-BY01 HIFU Tumour Therapy Equipment, Beijing Yuande Medical Instruments Company, Beijing, China) with late-stage pancreatic carcinoma. Their appetite, sleeping and mental health improved, and 5/15 patients gained weight. The tumours shrank in three cases and showed no change in the remaining 12. No serious complications (such as acute pancreatitis, damage to the stomach or intestines) were seen, and amylase levels were unaltered in 13/15 patients. Mild pancreatitis was observed in two cases (***parameters:*** *Power = 0.5–1.6 kW*) [130]. Another report was published in 2005 in which eight patients with unresectable pancreatic tumours of 5 to 8 cm in diameter experienced alleviation of their chronic pain (follow-up time 2–17 months), tumour shrinkage (20–70%) and no local complications. In two patients, regression of their metastasis was noted after ultrasound-guided HIFU treatment (Chongqing Haifu system) using a 0.8 MHz, 12 cm diameter transducer, with a focal region of 9.8 mm along the beam axis and 1.3 mm transversely (***parameters:*** *Power = 10–15 kW/cm^2^*) [131]. A retrospective study of 89 HIFU-treated advanced pancreatic cancer patients also showed that patients experienced effective pain relief (>80%), a partial response (~14%), subcutaneous sclerosis (6%), superficial skin burns (3%). One asymptomatic pancreatic pseudocyst case was seen [132].

Overall survival figures for pancreatic cancer patients have shown improvement after HIFU treatment. In 2011, the treatment of 13 stage III and 27 stage IV pancreatic cancer patients with ultrasound-guided HIFU (Chongqing Haifu Tech, Chongqing, China) resulted in median overall survival of 10 months for patients with stage III disease, and 6 months for patients with stage IV disease. The one-year survival rate as a whole was 30% [133]. In the same year, a similar 1-year survival rate was reported by Sung and colleagues after treatment of 46 stages III and IV pancreatic cancer patients [134], and in 2013, by Gao and colleagues after treatment of 39 patients with local advanced pancreatic cancer [135]. Both studies used the JC HIFU system for their ablative focused ultrasound treatments. In a study of 689 patients with unresectable pancreatic cancer, a statistically significant improvement in median survival for the HIFU group (ultrasound-guided HIFU (Chongqing Haifu Technology Co., Ltd)) (7.1 months) was seen compared with the non-HIFU group (5 months). In addition, there was a significant improvement in the overall survival of patients when using repeated HIFU treatments (8.6 months) compared to single HIFU treatments (6.8 months) [136]. Ultrasound-guided HIFU (JC200) has also been used as preoperative adjuvant therapy for borderline resectable pancreatic cancer. Here, the mean tumour ablation volume was ~60 ± 25%, and 28 of the 30 patients treated were able to have their tumours resected 7–9 days after HIFU treatment [137].

In Europe, the first report of patients treated with ultrasound-guided HIFU with palliative intent came from Italy. Six patients with pancreatic tumours of diameter 4.6 ± 1.4 cm were treated with HIFU ablation (JC HIFU system) using a 0.8 MHz, 20-cm diameter transducer, with a focal length of 15 cm. For these treatments, the patients followed a strict diet for 3 days and degassed water balloons were used to apply abdominal pressure to push the bowel out of the acoustic path. In all but 1 patient, the procedure was carried out with no complications and the 1-year survival rate and median survival after HIFU treatment were approximately 43% and 7 months, respectively (***parameters:*** *Energy = 793,168 ± 704,778 kJ*) [138]. In a second study, from Italy, MR-guided HIFU treatments of patients with unresectable pancreatic tumours were deemed to have been safe, and in five of the six patients, no tumour regrowth was seen [139]. In Germany, a prospective study showed that 50 patients with late-stage pancreatic cancer benefited from significant pain relief (>80%), tumour volume reduction (~60%) 6 months after ultrasound-guided HIFU treatment (JC system). Their median overall survival was 16.2 months from diagnosis suggesting a significant survival benefit (***parameters:*** *Power = 300 W for tumours < 4 cm in diameter, else Power = 400 W, Energy = 17 ± 12 kJ/mL*) [140]. Finally, in two different European centres (Germany and Bulgaria), not only pain relief but also improved physical, emotional and social parameters were reported for 80 pancreatic cancer patients treated with ultrasound-guided “thermal” HIFU. For these ultrasound-guided treatments, the JC TTS HIFU system was used allowing a 1 cm safety margin (***parameters:*** *Power = 100–400 W*) [141]. Collectively, these studies showed the feasibility and safety of treating pancreatic cancer patients with HIFU.

### 3.2. HIFU in Combination with Traditional Oncologic Interventions

HIFU has been combined with traditional oncologic interventions, such as chemotherapy and radiotherapy, to treat pancreatic cancer patients. Pain relief and minimal side effects have been observed in the majority of patients [142,143,144,145,146,147,148,149,150,151]. When oxaliplatin and gemcitabine were combined with HIFU (HIFUNIT-9000, Shanghai Aishen Sci-Tec Co., Shanghai, China), the 1-year overall survival rate of patients was 60% with no significant difference between patients at different stages of disease (middle and advanced pancreatic cancer) [145]. In a phase II trial, three treatments of HIFU (HIFUNIT-9000) repeated every 28 days were given to pancreatic cancer patients concurrently with gemcitabine. Out of the 37 patients treated, complete response was seen in two and partial response in 15 patients. Pain relief was reported in ~80% of patients and 1-year survival rates reached 50% [143]. In addition, partial response in four patients and stabilised disease in 22 was seen, when 16 stage III and 14 stage IV, pancreatic cancer patients were treated with HIFU (FEP-BY02 HIFU system) and chemoradiotherapy. Adverse events were reported in 10% of patients, including two patients with pseudocyst formation and one patient with mild pancreatitis [148]. A recent retrospective study showed that treatment of patients with unresectable PDAC with ultrasound-guided HIFU (Chongqing, China) and gemcitabine (347 patients) had an improved overall survival (7.4 vs. 6.0 months) and 1-year survival rates (21% vs. 14%) compared to patients who received gemcitabine only (176 patients). In addition, no severe complications were observed in patients receiving HIFU [142]. In another study with a “control” arm, a statistically significant improvement in 6-month survival, clinical benefit and pain remission rate was seen when 23 patients with unresectable pancreatic tumours were treated with ultrasound-guided HIFU (Chongqing, China) in combination with gemcitabine compared to 22 patients treated with gemcitabine alone (survival: 74% vs. 41% respectively, clinical benefit: 70% vs. 36%, respectively, pain remission rate: 65% vs. 32% respectively) [146].

In 2015 a report of a Spanish study of treatment of 43 patients with stage III or IV locally advanced pancreatic cancer with ultrasound-guided HIFU (JC HIFU system) and gemcitabine-based palliative chemotherapy demonstrated complete responses in 25% of patients, the overall median survival of 16 months, and a 33.5% survival of ~4 years. No deaths, emergency surgical procedures, large blood vessel rupture, nor gastrointestinal perforation were seen [149]. In 2016, a series of reports from a German study showed tumour reduction in approximately 60% of 13 patients 3 months later and complete or partial pain relief in 77% of patients after ultrasound-guided tumour ablation with the JC HIFU system (***parameters:*** *Treatment time = 114.5 ± 29 min, Sonication time = 19 ± 7 min, Power = 344 ± 72 W*) [150]. In a second published study, this German group showed that HIFU treatment of pancreatic tumours resulted in tumour devascularisation (***parameters:*** *Treatment time = 110.5± 30.7 min, Sonication time = 18.4 ± 7.6 min, Energy = 386 ± 256 kJ*) [151].

### 3.3. Clinical Case Studies

Case study reports have also demonstrated successful HIFU treatments. A 47-year-old patient with locally advanced unresectable pancreatic adenocarcinoma tumour in the body of the pancreas (4.0 cm × 3.2 cm) but with no metastasis in other organs was treated with 27 HIFU ablation sessions (HIFU-2001; Shanghai Jiaotong University, Shanghai, China) to the tumour and nodal disease, in addition to six cycles of gemcitabine and capecitabine. The patient recovered fully after these treatments [152]. Ultrasound-guided HIFU (JC Haifu system) in combination with gemcitabine led a 61-year-old patient with recurrent pancreatic cancer to stable disease and complete pain relief (***parameters:*** *Energy = 12 kJ/mL*) [153]. Recently, tumour shrinkage and pain reduction were reported in a 63-year-old patient, whose unresectable pancreatic carcinoma was progressing after gemcitabine and radiotherapy. Sadly, for this patient, despite the seven HIFU treatments (FEP-BY 02 HIFU Therapeutic System), they succumbed to their disease 3 years and 6 months later [154].

### 3.4. Complications of HIFU Treatments in Pancreatic Cancer Patients

No major complications resulting from HIFU treatments in pancreatic cancer patients have been reported. In 2011, third-degree burns and fistula formation between the tumour and duodenum were reported in a subset of 35 patients treated with ultrasound-guided HIFU (JC system) [155]. A prospective study of 87 patients with unresectable locally advanced pancreatic cancer treated with ultrasound-guided HIFU (HIFUINT-9000 system) identified 25 patients with adverse events, including fatigue (14 patients), abdominal pain and fever (7 patients), nausea and rash (5 and 4 patients, respectively) [156]. In 2019, minor complications were seen in 43/86 pancreatic cancer patients treated with ultrasound-guided HIFU (JC Chongqing Haifu system), including transient fever, abdominal pain, skin burn and amylase elevation in some cases [157]. Ning and colleagues reported adverse events, including increased serum and urinary amylase, fever and intestinal obstruction in a subset (<8%) of their HIFU-treated patients [142]. Abnormal amylase levels, gastrointestinal dysfunction and one incidence of obstructive jaundice have also been reported [158]. Gastrointestinal bleeding and severe pancreatitis in one patient and grade III skin burning in 3 patients was seen when 43 patients with stage III and IV pancreatic cancer were treated with systemic chemotherapy and ultrasound-guided HIFU [149]. Other side effects included peri-procedural hypothermia (<35 °C) and hyperthermia (>37.5 °C), which were reported in 10/71 ultrasound-guided HIFU-treated pancreatic cancer patients using the JC HIFU therapeutic system. In this study 1 s sonications were delivered at each focal point with 3 s intervals, and greyscale changes in the target area were deemed to demonstrate effective ablation. Hypothermia during the interventional procedure was noted in eight patients. This was resolved without the need to send the patients to the post anaesthesia care unit in 7/8 patients. Hyperthermia was seen in two patients. In most patients, a maximum difference of 1.3 °C in body temperature was seen and no other major complications were seen (***parameters:*** *Treatment time = 124 ± 45 min, Sonication time = 936 ± 366 s, Energy = 343 ± 151 kJ*) [159].

In contrast, a retrospective investigation of the endocrine and exocrine function of the pancreas in 59 HIFU-treated patients with advanced pancreatic cancer found no change in amylase or glucose levels before or after treatment [160]. In another study, designed to investigate the effects of treatment in the peri-pancreatic blood vessels of 15 ultrasound-guided HIFU treated patients (Chongqing system), 13 of these had blood vessels surrounded or invaded by the tumours, but no significant differences in postoperative hemodynamic data and no negative effects on blood vessels were seen [161]. Furthermore, blood vessel patency remained unchanged after HIFU therapy in 47/50 pancreatic cancer patients [162]. Using the FEP-BY02 system ultrasound-guided HIFU, there was reduced incidence of abdominal pain and fever when a “low power” HIFU approach (100–300 W) was used instead of “traditional” HIFU (400–1000 W) in 38 stage III patients [163] and 55 metastatic patients [164] with locally advanced pancreatic tumours. A question that is always asked about HIFU treatments is whether they can increase the metastatic rate. While there is no clinical evidence that this occurs, preclinical studies have addressed this concern. In one preclinical study, therapeutic focused ultrasound reduced the risk of metastatic spread in a human orthotopic BxPC-3 tumour model grown in mice. When these tumours were treated with focused ultrasound haematogenous metastasis (as detected by flow cytometry of circulating tumour cells passing through a slit in the artery of the subject’s ear), was reduced in the ultrasound treated group compared to the untreated group, while tumour growth was suppressed (***parameters:*** *Power = 47.92 W, I_sa_ = 1134 W/cm^2^, pulse duration = 500 ms*) [165].

To improve the safety and efficacy of HIFU treatments, methods of compensating for respiratory motion and improving thermometry have been developed, e.g., by fluid-filling the digestive tract surrounding the pancreas of patients [166,167,168,169,170]. For example, simulation data in seven patients showed that the air in the digestive tract and its proximity to the pancreas led to errors in temperature of more than 10 °C due to differences in the magnetic susceptibility between the two tissues and peristaltic motion. By filling the digestive tract with fluids with high Manganese content and favourable relaxation times, simulated temperature precision was improved to be within 2 °C. This method also improved the reconstruction of the background and MR imaging by minimising local field inhomogeneities.

### 3.5. Clinical Use of “Mechanical” Therapeutic Focused Ultrasound for the Treatment of Pancreatic Cancer Patients

So far, “mechanical” focused ultrasound treatments have received less clinical attention than “thermal”. The efficacy of the combination of concurrent gemcitabine and ultrasound-guided pHIFU (pHIFU FEP-BY™ HIFU unit) for the treatment of three patients with unresectable pancreatic cancer was compared to that of pHIFU treatments alone (nine patients). For the three patients who were successfully treated with gemcitabine and pHIFU, their overall survival ranged from 11 to 26 months. These three patients received a total of 32 treatments including six with pHIFU. Side effects included skin burns and mild abdominal pain, which were ultimately resolved. For the pHIFU-only treated patients, one case of pancreatitis was reported, and their median overall survival was ~10 months. Two cases of heat-induced subcutaneous sclerosis were also seen when more than 1000 J/lesion was delivered. In the 1st case, the injury was resolved within 4 weeks without treatment, but in the 2nd the injury remain unresolved 4 months later (***parameters:*** *Energy = 0.5–1 kJ, d.c. = 50%, prf = 3.3 Hz, pulses = 50–70*) [171].

### 3.6. Drug Delivery and Sonodynamic Therapy Studies in Preclinical Pancreatic Cancer Models

Several preclinical studies have shown an improvement in drug delivery and anti-cancer effects in pancreatic tumours using pHIFU, sonoporation or sonodynamic therapy. In 2014, human Panc-1 tumours grown in nude Balb/c subjects were treated with “low” and “high” power focused ultrasound using an US-guided 1.1 MHz transducer (Alpinion VIFU-2000, Bothell, WA, USA). The combination of “low power” therapeutic focused ultrasound and gemcitabine induced higher levels (approx. ~1.5 fold) of necrosis, and apoptosis than unsonicated gemcitabine only treated, or “high power” therapeutic focused ultrasound treated subjects (***“low power” parameters:*** *P– = 3.2 MPa, d.c = 5%, prf = 1 Hz, duration = 30 s, I_sata_ = 5.9 W/cm^2^, I_spta_ = 21.8 W/cm^2^, Energy = 15 W s, **“high power” parameters:** P– = 3.2 MPa, d.c. = 50%, prf = 40 Hz, duration = 10 s, I_sata_ = 58.6 W/cm^2^, I_spta_ = 218.87 W/cm^2^, Energy = 50 W s*) [172]. In addition, in GEMM KPC mouse models, pHIFU exposures induced high levels of cavitation activity, caused the disruption of the stroma and enhanced doxorubicin concentration in the tumours by up to 4.5-fold compared to sham-exposed subjects. The mechanism of doxorubicin increase in the tumours was suggested to be passive diffusion into the permeabilized tumours [173]. In the same model, a 23-fold increase in the drug concentration in the tumours was seen when temperature-sensitive liposomal doxorubicin in combination with MR-guided HIFU-induced hyperthermia was used, compared to sham-exposed subjects (liposomal doxorubicin only-no hyperthermia treatment). For the pHIFU treatments, a clinical MR-HIFU system (Sonalleve V1, Philips, Vantaa, Finland) on a clinical MRI scanner (Achieva 3 T, Philips) was used. In contrast, when subjects were exposed to the MR-guided hyperthermia and the non-liposomal doxorubicin formulation only a 2-fold increase in intratumoural drug concentration was seen compared to the sham-exposed subjects (subjects treated with drug only, no hyperthermia treatment). It should be noted that in the absence of hyperthermia treatment of the tumours, their non-liposomal doxorubicin concentration was approximately eight times higher than that when liposomal doxorubicin was used (***parameters:*** *f = 1.2 MHz, Power = 7 W, d.c = 100%, T = 41.2 ± 1.3 °C*) [174]. Additional studies have shown that the delivery of gemcitabine to pancreatic tumours was enhanced by sonoporation in human pancreatic MiaPaCa-2 tumours grown orthotopically in mice [175], while the combination of sonodynamic therapy with various anti-cancer compounds, including 5-aminolevulinic acid [176,177], 5FU [178,179], antibiotic and root extracts [180], gemcitabine [181], nanocomposites able to generate reactive oxygen species or nitric oxide [182,183,184], radiation [185] and epirubicin [186], have provided proof of concept for the intriguing potential that sonodynamic therapy has in pancreatic cancer.

## 4. The Immunological Effects of Therapeutic Focused Ultrasound in Pancreatic Cancer

The benefits of immunotherapy have failed to materialise in patients with locally advanced or metastatic pancreatic cancer as has been shown in clinical trials involving anti-CTLA, anti-PD-1 and anti-PDL1 antibodies alone or in combination with gemcitabine, gemcitabine and nab-paclitaxel, anti-CCR4, tyrosine kinase inhibitors, anti-CXCR4, oncolytic viruses and vaccines (GVAX) [187,188,189,190,191,192,193,194,195,196,197,198,199,200]. For this reason, efforts are underway to discover new treatment modalities that can be used in combination with immunotherapies. Focused ultrasound could enhance the effects of immunotherapy in pancreatic cancer by destroying cancer cells, releasing tumour-specific antigens, DAMPS, and inducing ICD, thus attracting TIL, activating APC, including DC, and increasing the permeability of pancreatic tumour stroma thus allowing more immune-therapeutics to access the tumours (Table 3).

The first clinical study to report the immunological effects of HIFU in the pancreas was published in 2002. Increased natural killer cell activity and increases in the abundance of TIL were seen after “thermal” HIFU treatment. In this study, 15 patients with an average age of 62 years and tumour size on average of 5.6 cm participated and were treated with ablative “thermal” HIFU (FEP-BY01 system). The immune effects of these treatments were studied in the peripheral blood of 10 patients. Statistically significant increases of 25% in NK cells were seen, as well as non-statistically significant differences in CD3^+^ cells and CD4^+^ cells compared to before HIFU treatment (***parameters:*** *Power = 0.5–1.6 kW, Beamed power = 1000–1400 kW*) [130]. Recently, immune and anti-cancer effects of thermal HIFU in pancreatic cancer have been demonstrated in 100 patients with locally advanced inoperable pancreatic tumours in a retrospective clinical study. Patients were treated with the ultrasound-guided “thermal” HIFU (JC TTS, Haifu Medical Technology, Chongqing, China). The system consisted of a treatment table with an integrated high power focused US-therapy transducer (diameter 20 cm, focal length 15 cm, frequency 0.8 MHz), Significant reduction in tumour volumes of patients were seen from 6 weeks to 12 months after thermal ablation, as was acute induction of inflammation, including an increase in leukocytes 2, 5 and 20 h after thermal ablation, compared to baseline. Furthermore, non-statistically significant increases in IL-6 compared to baseline were seen 20 h after ablation (***parameters:*** *Treatment time = 124 ± 46 min, Sonication time = 932 ± 374 s, Energy = 343.9 ± 156.2 kJ, Power = 366 ± 91 W, Energy/Volume = 12.8 ± 4.5 kJ/mL*) [203]. In a case study of a 48-year-old patient with unresectable metastatic pancreatic cancer, they were given “thermal” HIFU with the JC HIFU system as a palliative therapy after poor response to gemcitabine/erlotinib and subsequently to FOLFOX (folinic acid, oxaliplatin and 5-FU). The patient then continued with FOLFOX. Decreases in tumour and metastatic lesion sizes were seen 12 months after HIFU, suggesting the possible induction of the abscopal effect (***parameters:*** *Power = 103 W, Sonication duration = 752 s, 1st hyperechoic signal detected = 150 s.*) [201]. In another clinical case, a 74-year-old patient presented with anaplastic pancreatic carcinoma. This patient had a 3 cm pancreatic body lesion which progressed to 4.6 cm after chemotherapy, and multiple pathological lymph nodes in the retroperitoneal region were seen. At that point, the patient underwent “thermal” HIFU ablation on the pancreatic lesion using the JC HIFU system (f = 0.8 MHz, focal length = 15 cm, transducer diameter = 20 cm) as a way of providing a locoregional palliative procedure. The ablation was evaluated with power Doppler, lesions were separated vertically by 5 mm. Imaging was performed using the MyLab70 imaging device (Esaote, Genova, Italy) (1.0 to 8.0 MHz imaging probe). A CT scan performed the day after treatment revealed a 5.6 cm pancreatic lesion and paraaortic lymphadenopathies. After HIFU the patient received five more cycles of chemotherapy. The patient became asymptomatic after HIFU, and a restaging CT scan 4 months later revealed a ~30% reduction in the size of the pancreatic head lesion and a 75% reduction in the size of the left para-aortic lymph node lesions suggesting the induction of an abscopal effect [202].

In preclinical studies, histotripsy treatment of approximately 60–75% of subcutaneous murine Pan02 tumours with an ultrasound-guided transducer induced a cavitation cloud, confirmed by ultrasound imaging, tumour destruction and a decrease in tumour size. No changes in immune cells in the microenvironment of the tumours were seen 24 h and 7 days after histotripsy, and then a decrease in macrophages (percentage of CD45^+^ cells) and regulatory T cells (percentage of total cells) and an increase in DC (as a percentage of CD45^+^ cells) was seen 14 days after histotripsy treatments. The same authors also showed the release of DAMPS, including HMGB1 release, after the treatment of the Pan02 cells in vitro with the same histotripsy parameters (***parameters:*** *f = 1 MHz, 8 elements, f-number = 0.68, focus = 0.98 × 0.93 × 3.9 mm, pulses < 2 cycles, prf = 250 Hz*) [204]. Sonodynamic therapy has also shown pro-immune effects in preclinical models of pancreatic cancer. When hypoxia-alleviating polymethacrylate-coated CaO_2_ nanoparticles were administrated systemically in animals bearing syngeneic bilateral KPC tumours, sonodynamic therapy with a SP100 Sonidel sonoporator (Sonidel Ltd, Dublin, Ireland) increased T_cytotoxic_ and decreased T_regulatory_ in both target and off-target tumours and induced the abscopal effect in off-target tumours (***parameters:*** *f = 1 MHz, I_satp_ = 3 W/cm^2^, d.c. = 30%, prf = 100 Hz*) [205]. In another recently published study, microbubble mediated sonodynamic therapy controlled tumour growth and induced an abscopal effect in a bilateral syngeneic KPC tumour mouse model. Here, bilateral tumours were established in mice and one of them was treated with ultrasound during systemic administration of a microbubble-Rose Bengal conjugate (MB-RB) and injection of anti-PD-L1. A significant decrease in both the target and off-target tumour volumes of animals treated with sonodynamic therapy and anti-PD-L1 was seen when compared with sham-exposed animals. Furthermore, CD4^+^ T cells and T_cytotoxic_ cell infiltration in the off-target tumour of the treated animals was increased by the sonodynamic therapy (***parameters:*** *f = 1 MHz, d.c. = 30%, prf = 100 Hz, P– = 0.48 MPa, mechanical index = 0.48, Sonication duration = 3.5 min during and 30 min after injection*) [206]. Focused ultrasound has also been combined with immune checkpoint inhibitors to treat orthotopic murine pancreatic KPC tumours with the Alpinion VIFU200 platform. When the subjects were treated with pHIFU, an improvement in the survival of animals treated with the combined pHIFU and ICI treatment (anti-CTLA-4 and anti-PD-1 antibodies (200 μg/dose) injected in the intra-peritoneum every 3 days) was seen compared to the survival of the control subjects, and those of the pHIFU and ICI only treated groups. This improvement was associated with an increase in T_cytotoxic_, IFNγ^+^ T_cytotoxic_ cells, and the ratio of these cells to T_regulatory_ and MDSC in the tumours of the animals treated with pHIFU and ICI compared to the control animals. These results suggested that treatment of pancreatic tumours with pHIFU can enhance ICI anti-cancer therapy by changing the immune contexture of tumours (***parameters:*** *f = 1.5 MHz, P– = 17 MPa, prf = 1 Hz, d.c. = 1%, pulse duration = 10 ms*) [112].

## 5. Conclusions and Future Directions

Despite encouraging clinical results and the lack of severe adverse effects from therapeutic focused ultrasound treatments, no randomised controlled trial to test whether focused ultrasound can provide a significant survival benefit to patients with pancreatic cancer has ever been undertaken. The FDA approval of ICI for a small minority of pancreatic cancer patients (~1%) with high microsatellite-instability in their genomes [60,207] provides hope that treatment of pancreatic cancer patients with immunotherapies can be possible if focused ultrasound treatment can render immunologically “cold” tumours “hot”. In addition, evidence exists that chemoradiotherapy treatments can be used without impairing immune responses, thus leading the way for their use in combinatorial treatment regimens involving therapeutic focused ultrasound [208,209,210]. Several clinical trials and studies are underway to test the combination of immunotherapy (ICI, MHC-II agonists, CD40 and TLR agonists, oncolytic viruses, adoptive T cell therapies and vaccines) with these oncology interventions and reviews are available [211,212].

Stiff pancreatic tumours are excellent candidates for treatment with physical modalities, such as therapeutic focused ultrasound. Strong evidence is now in place, both preclinically and clinically, that therapeutic focused ultrasound has a significant benefit in the treatment of pancreatic cancer. Progress is hindered, in part, by the high cost of some immunotherapies, although ICI antibody patent release in the following years would be expected to lower the associated costs. Furthermore, technological developments (e.g., thermometry, motion compensation, software user interfaces, and endoscopic transducers) are expected to lead to whole tumour ablation in most cases. The development of mechanical HIFU for clinical use will offer greater pro-inflammatory effects in patients, as has been shown preclinically. The establishment of an ultrasound thermal and cavitational dose will improve our understanding of the bioeffects of focused ultrasound and provide the capability for clinicians to standardise treatment between different centres. Ultimately the rapid growth in the use of therapeutic focused ultrasound in the clinic will continue, and in the following years, its increased use in combinatorial treatment protocols for pancreatic cancer patients will be seen.

## Figures and Tables

**Table 1:** Examples of frequencies and intensities used in focused ultrasound treatments with therapeutic intent.

Treatment Type	Frequency	Treatment Parameters (Intensity)	Therapeutic Intent	Translational Stage	Ref.
Low intensity focused ultrasound—continuous exposures	1.5 MHz	0.5 W/cm^2^ for 5, 10 and 25 min /day for 25 days	Fracture repair	In vivo preclinical	[101]
Thermal (Hyperthermia)	1–3 MHz	0.3–2 W/cm^2^ until temperature reached 45 °C	Tumour sensitisation in combination with radiotherapy/chemotherapy	Clinical	[102]
Thermal (ablation)	0.8 MHz	5 kW/ cm^2^	Tumour ablation	Clinical	[105]
Mechanical (pHIFU)	1.15 MHz	2.7 kW/cm^2^ spatial average temporal peak	Tumour ablation	In vivo preclinical	[106]
Mechanical (Histotripsy)	2 MHz	14 kW/cm^2^	Tissue atomisation	In situ preclinical	[103]
Low intensity focused ultrasound—pulsed exposures	42 kHz	0.15 W/cm^2^	Cell proliferation	In vitro preclinical	[104]

**Table 2:** Seminal studies showing focused ultrasound-induced immune effects in tumours of non-pancreatic origin.

Therapeutic Focused Ultrasound	Study and Tumour Type	Treatment Parameters	Immunologic Effect	Ref.	Year
Thermal hyperthermia	preclinical in vitro, colon cancer cells	TID: 60–120 CEM_43_	DAMP release and induction of ICD	[80]	2021
Thermal ablation	preclinical in vitro, lung and ovarian	TID >> 240 CEM_43_	DAMP release and induction of ICD	[114]	2017
Thermal ablation	preclinical in vivo, murine hepatocellular tumours	f: 9.5 MHz, acoustic power: 5 W, exposure time:220 s	Increased T_cytotoxic_ activity, IFNγ and TNFα, DC maturation	[111,115]	2010, 2012
Thermal ablation	preclinical, melanoma tumours	TID >> 240 CEM_43_ in combination with immunotherapy	Antigen cross-presentation, IFNγ release	[116]	2018
Thermal ablation	clinical, osteosarcoma, hepatocellular and renal cell carcinomas, breast cancer	acoustic focal peak intensities: 5–20 kW/cm^2^	Increases in various immune cells in the blood and tumours including T cells and APC	[105,109,110]	2004, 2009
Mechanical pHIFU	preclinical, in vivo colon adenocarcinoma, prostate tumours	d.c.: 2%, P–: 10–12.5 MPa	DC maturation/accumulation of tumour-specific IFNγ-secreting cells	[117,118]	2007, 2012
Mechanical pHIFU	preclinical, in vivo melanoma, breast tumours	P–: ~6 MPa, d.c.: 10%, prf: 5 Hz,	Various pro-immune anti-tumour effects in the tumours, TDLN and spleen of subjects	[119,120,121]	2019, 2021, 2020
Mechanical histotripsy	preclinical in vivo, melanoma tumours expressing cancer antigen	f: 1 MHz, P–: 30 MPa, prf: 100 Hz	Stimulating cancer-specific lymphocyte responses	[108]	2020
Mechanical histotripsy	preclinical, in vitro breast cancer cells	10 ms-long pulses, P–: 14 MPa, prf: 1 Hz, d.c.: 1%,	DAMP and cytokine release, induction of ICD	[122]	2019
Mechanical pHIFU	preclinical in vivo neuroblastoma tumours	f: 1.5 MHz, P–: 14 MPa, 13.33-ms long pulses, prf: 1 Hz,	Induction of systemic inflammation	[123]	2020
Mechanical histotripsy	preclinical in vivo renal cell carcinoma	P–: 17–20 MPa, 10 ms duration, prf = 1 Hz	Tumour infiltration of T_cytotoxic_ cells	[124]	2019
LOFU pulsed exposures	preclinical in vivo, colon tumours	P–: 1.4 MPa, 100 ms long pulses, prf: 1 Hz	Increases in T_cytotoxic_ cells T_cytotoxic_/T_regulatory_ ratio	[125]	2012
LOFU continuous exposures	preclinical in vivo, melanoma tumours	P–: 3 MPa, d.c.: 100%	Reversion of T cell tolerance and anergy	[113]	2016
LOFU pulsed exposures	preclinical in vivo, murine brain	P–: 0.3 MPa, 10-ms bursts, 1% duty cycle	Sterile inflammation	[126,127]	2018 2017

**Table 3:** Complete list of published studies showing the immunological effects of therapeutic focused ultrasound in pancreatic cancer.

Year	Tumour Type	Focused Ultrasound Parameters	Immunologic Effect	Ref.
2002	Clinical study—thermal ablation	Input power: 0.5–1.6 kW Beamed power: 1–1.4 MW	Increases NK cells, T cells, CD4^+^ cells	[130]
2015	Case study—thermal ablation	Power: 103 W, treatment time: 752 s	Abscopal effect	[201]
2016	Case study—thermal ablation	f: 0.8 MHz	Abscopal effect	[202]
2021	Clinical retrospective—thermal ablation	f: 0.8 MHz, power: 366 W, energy/volume: 12.8 kJ/mL	Increases in cytokines (IL-6) and leukocytes	[203]
2021	Preclinical histotripsy in murine subcutaneous Pan02 tumours	f: 1 MHz, prf: 250 Hz, pulses < 2	Decreases in macrophages, regulatory T cells, increases in dendritic cells, release of DAMPS	[204]
2021	Preclinical sonodynamic therapy in syngeneic KPC bilateral tumours	f: 1 MHz, Isatp: 3 W/cm^2^ d.c.: 30% prf: 100 Hz treatment duration:3.5 min	Abscopal effect, increased T_cytotoxic_ and decreased T_regulatory_ cells	[205]
2021	Preclinical sonodynamic therapy—syngeneic KPC tumours	f: 1 MHz, d.c.: 30%, prf: 100 Hz, P–: 0.48 MPa, in combination with microbubbles/anti-PD1	Increases in T_cytotoxic_ and CD4^+^ T cells in off-target tumours	[206]
2021	Preclinical pHIFU—murine syngeneic orthotopic KPC tumours	f: 1.5 MHz, d.c: 1%, prf: 1 Hz, P–: 17 MPa, 10 ms long pulses in combination with anti-CTLA-4/anti-PD-1	Increased T_cytotoxic_ cells, IFNγ^+^ T_cytotoxic_ cells and the ratio of these cells to T_regulatory_ and MDSC in the tumours	[112]
